# Targeting the MDSCs of Tumors In Situ With Inhibitors of the MAPK Signaling Pathway to Promote Tumor Regression

**DOI:** 10.3389/fonc.2021.647312

**Published:** 2021-03-18

**Authors:** Jiayun Yu, Hanwen Li, Zongliang Zhang, Weimin Lin, Xiawei Wei, Bin Shao

**Affiliations:** ^1^ State Key Laboratory of Oral Diseases, National Clinical Research Center for Oral Diseases, West China Hospital of Stomatology, Sichuan University, Chengdu, China; ^2^ Department of Radiotherapy, Cancer Center, State Key Laboratory of Biotherapy and Cancer Center, Laboratory of Aging Research and Cancer Drug Target, National Clinical Research Center for Geriatrics, West China Hospital, Sichuan University, Chengdu, China

**Keywords:** myeloid-derived suppressor cells, mitogen-activated protein kinases, tumor microenvironment, apoptosis, lymphocyte

## Abstract

Myeloid-derived suppressor cells (MDSCs) are one of the major components of the tumor microenvironment. Evidence has shown differences in the functions and fates of MDSCs in the tumor tissue and the periphery. However, the exact mechanism that regulates MDSC function has not been completely clarified. In this study, we performed RNA sequencing of MDSCs derived from the spleen and tumor. Based on the results of our RNA-seq analysis, mitogen-activated protein kinases (MAPK) were significantly increased in tumor polymorphonuclear MDSCs (PMN-MDSCs) and monocytic MDSCs (M-MDSCs). Subsequently, 3 major MAPK pathways, including extracellular signal-regulated protein kinases (ERK), p38 and c-Jun NH2-terminal kinases (JNK), were studied to analyze the role of MAPKs in MDSCs. The ERK 1/2 inhibitor SCH772984 and the JNK inhibitor SP600125 significantly increased the apoptosis of both PMN-MDSCs and M-MDSCs *in vitro*. In addition, SCH772984 exerted a strong effect on inhibiting tumor growth. The flow cytometry analysis showed significant increases in the ratio of M1:M2 tumor-associated macrophages, meanwhile the number of CD4^+^, CD8^+^, CD4^+^CD69^+^ and CD8^+^CD69^+^ lymphocytes were increased after SCH772984 treatment. Our findings established the effect of MAPKs on the tumor microenvironment *via* MDSCs and may facilitate the development of new antitumor strategies.

## Introduction

The tumor microenvironment, which contains various types of cells, including endothelial cells, immune cells and fibroblasts, is an important factor of tumor development (1). It participates in a series of pathophysiological processes, such as proliferation, survival, and migration (2). Endothelial cells participate in tumor growth and protect the tumor from the immune system (3). Immune cells are associated with tumor development and the suppression of antitumor immune responses (4). Fibroblasts facilitate the migration of tumor cells into the blood and result in metastasis (5). Furthermore, tumor cells and their microenvironment create a suppressive environment that can enslave and mutate the infiltrated host cells, which makes them different from normal host cells. Due to the importance of the tumor microenvironment, the mechanisms involved in changing the tumor microenvironment might be potential therapeutic targets.

Myeloid-derived suppressor cells (MDSCs) are a series of immature myeloid cells that participate in pathological processes such as cancer, autoimmunity, and trauma (6, 7). MDSCs are categorized as PMN-MDSCs and M-MDSCs. In mice, MDSCs are characterized by the expression of both Gr-1 and CD11b. Low Gr-1 expression and CD11b^+^Ly6C^+^Ly6G^−^ are characteristics of M-MDSCs. On the other hand, PMN-MDSCs express Gr-1 at highs levels and are CD11b^+^Ly6C^−^Ly6G^+^ (8). In human patients, the antigens expressed on MDSCs are highly heterogeneous. Depending on the expression of CD11b and CD33 and the lack of mature markers of HLA-DR (9), M-MDSCs are defined as CD14^+^HLA-DR^lo^ or CD11b^+^CD14^−^CD33^+^CD15^−^ cells. Meanwhile, PMN-MDSCs are defined as CD11b^+^CD14^−^CD33^+^CD15^+^ cells (10). MDSCs play an important role in the immunosuppression of cancer (11) and are involved in tumor immune responses, including tumor immune escape (12). The immunosuppressive function of MDSCs are mediated by multiple molecular pathways, including programmed cell death protein 1 (PD-1)/programmed cell death-ligand 1 (PD-L1) checkpoint activation, transforming growth factor-β (TGF-β), and reactive oxygen and nitrogen species (ROS and RNS) (6,) (13, 14). In addition to affecting antitumor immunity, MDSCs also participate in the regulation of the tumor microenvironment (15). According to previous studies, MDSCs enhance the stemness of cancer cells and promote the epithelial-mesenchymal transition in the tumor microenvironment (16–18).

As mentioned above, MDSCs are abundant in tumor tissues. However, based on accumulating evidence, MDSCs in the peripheral tissues and the tumor site have different fates and functional specializations. In previous studies of the peripheral tissues from patients, the predominant MDSCs were PMN-MDSCs. PMN-MDSCs have a relatively moderate immunosuppressive function but are unable to differentiate into macrophages or dendritic cells (15). In addition, MDSCs are more prominent in tumor tissues and show a strong immunosuppressive function. A significantly lower level of phosphorylated STAT3 (p-STAT3) was recently detected in tumor-derived MDSCs than in peripheral MDSCs. An inhibitor of p-STAT3 only eliminated the activity of MDSCs in the peripheral tissues, such as the spleen, while MDSCs in tumors were not affected (19). The studies described above confirmed that the regulation of MDSCs in tumor and peripheral tissues might be different, and thus studies of the regulatory mechanism of MDSCs are very important.

Mitogen-activated protein kinases (MAPKs) participate in a wide range of cellular processes, including proliferation, apoptosis, differentiation and innate and adaptive immune responses (20). To date, 3 major groups of MAPKs have been identified in humans, including ERK, p38 and JNK (21). MAPKs have been proven to participate in many tumor processes. A number of drugs have been developed that specifically target this pathway and many clinical trials are ongoing. ERK1/2 inhibit cell apoptosis and result in prolonged cell survival (22). Dysfunction of ERK1/2 has been observed in various tumors, such as gastric adenocarcinoma, renal cell carcinoma and hepatocellular carcinoma. However, the roles of p38 and JNK in cancer are controversial. P38 is associated with p53-mediated apoptosis and the regulation of fibroblast transformation (23). On the other hand, increased levels of phosphorylated p38 have been detected in several tumors (20). JNK activation promotes tumor cell proliferation (24). However, JNK also induces cell death *via* DNA damage and is associated with autophagy (25).

In this study, we isolated MDSCs from spleens and tumors using Miltenyi magnetic beads. By performing a RNA-seq analysis, we screened the possible regulatory pathways that might participate in the functions of MDSCs. We specifically focused on the role of MAPKs in the regulation of MDSCs.

## Materials and Methods

### Cell Lines

LL2 cells were obtained from American Type Culture Collection (ATCC) and grown in RPMI 1640 medium plus 10% fetal bovine serum (FBS). Isolated MDSCs were grown in tumor explants supernatant (TES) conditioned medium (20% TES plus 80% RPMI 1640 medium plus 10% FBS). To generate tumor explants supernatant, LL2 tumor tissue were collected and cut into small pieces under sterile condition and cultured in RPMI 1640 medium plus 10% FBS for 24 hours.

### Animal Model and Drug Administration

The LL2 tumor model was established in 6-8-week-old female C57/BL6 mice, which were purchased from Vital River Laboratories (VRL). Mice were maintained in a specific pathogen-free animal facility for at least one week before our experiments. Initially, 1×10^6^ LL2 tumor cells were injected subcutaneously into the lateral side of the right thigh. When the size of the tumors reached about 50 mm^3^, the mice were randomly divided into 2 groups. The control group was treated with 200 μl of corn oil, and the SCH772984 group was treated with 12.5 mg/kg SCH772984 diluted in corn oil. Every other day, the mice received an intratumor injection of the drugs on the right side, left side and middle of the tumor. The tumor size was measured every two days. The tumor volume was calculated using the following formula: tumor volume (mm^3^) =length (mm) × width(mm)^2^ × 0.52.

### Antibodies and Flow Cytometry

An anti-CD4 FITC-conjugated monoclonal antibody (mAb), an anti-CD8 BV421-conjugated mAb, an anti-CD69 PE-conjugated mAb, an anti-CD11b FITC-conjugated mAb, an anti-Gr1 PE-conjugated mAb, an anti-F4/80 PE-conjugated mAb, an anti-CD206 AF647-conjugated mAb and the Annexin V-FITC/PI Apoptosis Detection kit were purchased from BD Biosciences (CA USA). The flow cytometry analysis was performed with a FACScan flow cytometer (Becton Dickinson) and Cell Quest software (BD Biosciences).

### Isolation of MDSCs From the Spleen and Tumor

Magnetic activated cell sorting was performed as described previously with minor modifications (26). Briefly, spleen and tumor tissue were collected and cut into small pieces. After an incubation with 20 ml of RPMI 1640 medium (serum free) containing 1 mg/ml of 280 U/mg collagenase I (Gibco) and 2 μl of 2 mg/ml DNase (Sigma) at 37°C for 1 hour, the tissue mixture was passed through a filter (70 μm). Then, the cell suspension was centrifuged at 300 g for 10 minutes at 4°C. The collected cells were resuspended and passed through the 70-μm filter again and then centrifuged on Histopaque^®^ -1077 (Sigma) at 400 g for 30 minutes. Subsequently, cells were collected from the interface and washed with RPMI 1640 medium. Gr-1^high^Ly6G^+^Ly6C^-^ (PMN-MDSCs) and Gr-1^dim^Ly6C^+^Ly6G^-^(M-MDSCs) cells were isolated from the collected cells through Miltenyi magnetic beads purification with anti-Gr-1 mAbs (RB6-8C5) and LS MACS columns according to the manufacturer’s protocol.

### RNA-Seq Analysis

PMN-MDSCs and M-MDSCs cells were isolated from the spleen and tumor tissue of the same mice as mentioned above (n=3). The isolated cells were treated with trizol and sent to Novogene Co., Ltd for RNA sequence. Kyoto Encyclopedia of Genes and Genomes(KEGG)enrichment, Gene Ontology (GO) enrichment, heatmaps were analyzed to upregulated and downregulated pathways.

### Treatment of MDSCs

Isolated PMN-MDSCs and M-MDSCs cells were cultured with TES conditioned medium as mentioned above and divided into 4 groups. In group A, cells were cultured with 1‰ DMSO (Sigma Co. Ltd) for 24h. Cells in group B were treated with TES and 3 nM LY2228820, a p38 MAPK inhibitor. Cells in group C were treated with TES and 3 nM SCH772984, an ERK 1/2 inhibitor. In group D, cells were treated with TES and 15 nM SP600125, a JNK inhibitor. All the inhibitors were purchased from Sigma Co. Ltd and dissolved in DMSO.

### Statistical Analyses

Data were obtained from two independent experiments. SPSS 23.0 software was used to analyze the data reported in this study. All data displaying a normal distribution are presented as means ± SD. One-way ANOVA with the least significance difference (LSD) *post hoc* test were used to compare data collected at individual time points. Survival data were analyzed with the log-rank test. P < 0.05 was considered statistically significant.

### Ethics Statement

The study was approved by the ethics committee of West China Hospital of Stomatology, Sichuan University.

## Results

### Differentially Expressed Genes in MDSCs From the Spleen and Tumor

**Figure 1:**
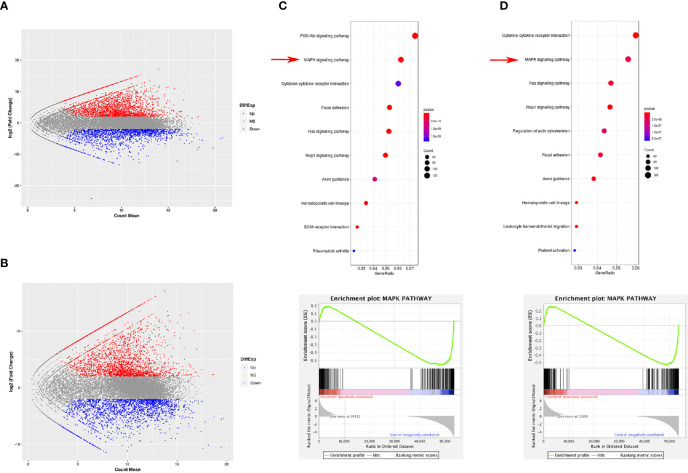
Gene expression difference between MDSCs from spleen and tumor. RNA sequencing was performed using MDSCs derived from the spleen and tumor using magnetic activated cell sorting to study the differences between PMN-MDSCs **(A)** and M-MDSCs **(B)** from the tumor tissue and spleen. The KEGG analysis showed statistically significant changes in the expression of genes involved in the MAPK pathway in both PMN-MDSCs **(C)** and M-MDSCs **(D)**.

RNA sequencing was performed using MDSCs derived from the spleen and tumor using magnetic activated cell sorting to study the differences between MDSCs from the tumor tissue and from other tissues, such as the spleen. After RNA sequencing of PMN-MDSCs and M-MDSCs in the spleen and tumor, we identified 9168 upregulated genes and 7827 downregulated genes in PMN-MDSCs (**Figure 1A**) and 7848 upregulated genes and 8967 downregulated genes in M-MDSCs (**Figure 1B**). In addition, MAPK signal pathway showed obvious changes in RNA sequencing. A comparative Kyoto Encyclopedia of Genes and Genomes (KEGG) analysis showed the statistically significant activation of the MAPK pathway in both PMN-MDSCs and M-MDSCs from tumors compared with the same cells from the spleen (**Figures 1C, D**).

### MAPK Inhibitors Promote MDSC Apoptosis

**Figure 2:**
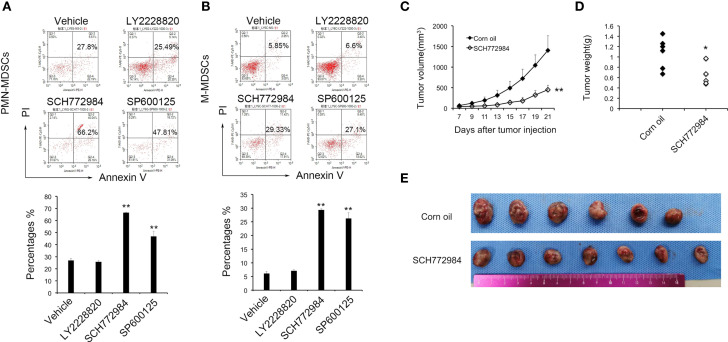
MAPK inhibitor induce the apoptosis of MDSCs and suppress tumor growth. LY6G **(A)** and LY6C **(B)** cells were treated with 3 nM LY2228820 and SCH772984, and 15 nM SP600125 *in vitro*. Based on the flow cytometry data, SCH772984 and SP600125 significantly increased MDSC apoptosis. SCH772984 induced apoptosis in a greater percentage of cells. Mice were treated with SCH772984 (12.5 mg/kg) twice a week after 1 × 10^6^ cells were injected. **(C)** In the SCH772984 group (n=7), tumor growth was significantly inhibited compared with the corn oil group (n=6). **(D)** The weight of the tumor in corn oil group was heavier than SCH772984 group. **(E)** Photograph of the tumor in each mouse, and the tumor volume of the SCH662984 group was smaller than the corn oil group. (*P < 0.05 and **P < 0.01).

To further investigate the role of MAPK in MDSCs, inhibitors of the three major MAPK pathways, P38, ERK and JNK, were used to determine the role of MAPKs in MDSCs. PMN-MDSCs and M-MDSCs cells were treated with 3 nM LY2228820 and SCH772984, and 15 nM SP600125 cultured with RPMI 1640 and TES. In the PMN-MDSCs cells from the Vehicle group, the apoptosis rate, which included early apoptotic and late apoptotic cells, was 26.80%. In PMN-MDSCs cells from the LY2228820, SCH772984 and SP600125 groups, the rates were 25.75%, 66.44% and 46.83%, respectively. In addition, in M-MDSCs cells from the Vehicle group, the rate of apoptosis was 6.07%. The apoptosis rates in LY6C cells from the LY2228820, SCH772984 and SP600125 groups were 7.06%, 29.28% and 26.17%, respectively. SCH772984 and SP600125 significantly promoted the apoptosis of both PMN-MDSCs (**Figure 2A**) and M-MDSCs (**Figure 2B**). SCH772984 induced a greater increase in apoptosis and was chosen for subsequent tests.

### The Antitumor Function of SCH772984

As mentioned above, SCH772984 exerted the most prominent effect on MDSC apoptosis among the three MAPK pathway inhibitors. The *in vivo* antitumor effect of SCH772984 was further confirmed in a mouse LL2 model. Tumor-bearing mice were treated with SCH772984 twice a week after 1×10^6^ LL2 cells were injected. The tumor volume in the SCH772984 group was significantly smaller than in the control group (**Figures 2C, E**). The average tumor weight in the SCH772984 group was 0.68 g, while the value of the control group was 1.08 g (p=0.017) (**Figure 2D**). The tumor growth curve, tumor weight and tumor volume suggested that SCH772984 significantly inhibited tumor growth.

### Function of the Tumor Microenvironment in Antitumor Effects

**Figure 3:**
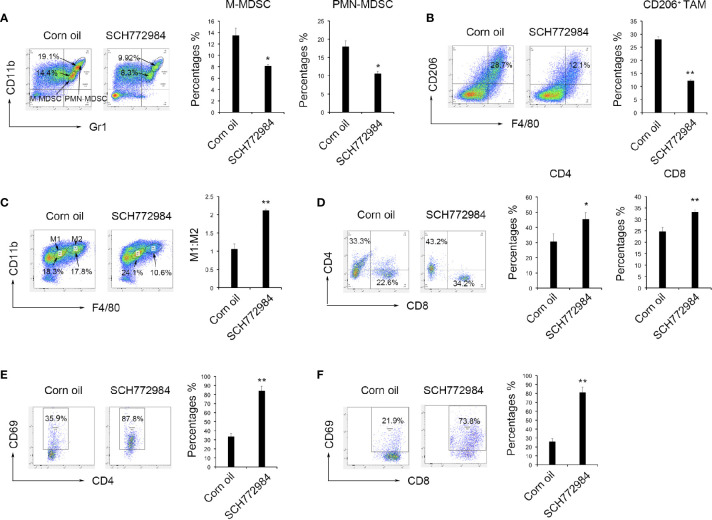
SCH772984 treatment alters the tumor microenvironment from immunosuppressive to immunostimulatory. Cells were prepared from mouse tumor tissues to study the change in the tumor microenvironment after treatment. **(A)** The numbers of both Gr1^+^CD11b^+^ M-MDSCs and Gr1^+^CD11b^+^ PMN-MDSCs in the tumors from mice decreased after the SCH772984 treatment. **(B)** The number of F4/80^+^CD206^+^ cells was also reduced in the SCH772984 group. **(C)** The ratio of M1:M2 tumor-associated macrophages increased in the SCH772984 group. **(D)** The numbers of CD4^+^ and CD8^+^ lymphocytes were increased in the tumors from mice. The numbers of both CD4^+^CD69^+^ **(E)** and CD8^+^CD69^+^ **(F)** lymphocytes isolated from tumors were significantly increased in mice from the SCH772984 group. (*P < 0.05 and **P < 0.01).

A single cell suspension was prepared from mouse tumor tissues to study the change in the tumor microenvironment after SCH772984 treatment. Based on the flow cytometry results, the numbers of both Gr-1^dim^Ly6C^+^Ly6G^-^ M-MDSCs and Gr-1^high^Ly6G^+^Ly6C^-^ PMN-MDSCs decreased after the SCH772984 treatment compared with the corn oil group (**Figure 3A**). In addition, the number of F4/80^+^CD206^+^ TAM was also reduced in the SCH772984 group (**Figure 3B**). The ratio of M1:M2 TAM increased in the SCH772984 group, suggesting that more M2-like macrophages transformed to M1 macrophages after treatment with SCH772984 (**Figure 3C**). The numbers of both CD4^+^ and CD8^+^ lymphocytes (**Figure 3D**) and CD4^+^CD69^+^ (**Figure 3E**) and CD8^+^CD69^+^ (**Figure 3F**) activated lymphocytes were significantly increased after the SCH772984 treatment. The results from the assays described above suggested a change in the immunosuppressive status of the tumor microenvironment.

### Lymphocytes in the Spleen and Lymph Nodes Were Activated

**Figure 4:**
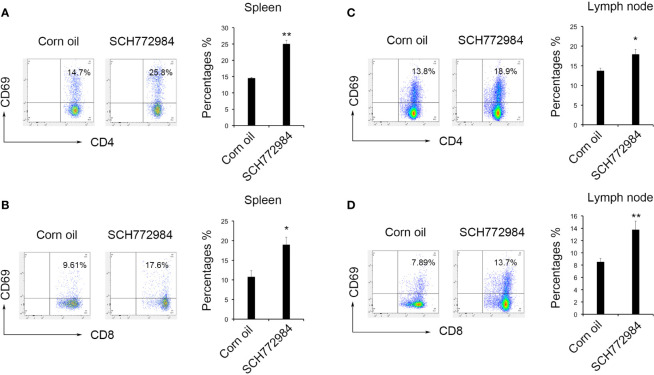
SCH772984 treatment improves T cells activation. Changes in the numbers of CD4^+^CD69^+^ **(A)** and CD8^+^CD69^+^ **(B)** cells in the spleen. The numbers of both CD4^+^ CD69^+^ and CD8^+^ CD69^+^ cells increased in the SCH772984 group. Changes in the numbers of CD4^+^CD69^+^ **(C)** and CD8^+^CD69^+^ **(D)** cells in lymph nodes. The numbers of both CD4^+^ CD69^+^ and CD8^+^ CD69^+^ cells increased in the SCH772984 group. (*P < 0.05 and **P < 0.01).

Changes in lymphocytes in the mouse spleens and lymph nodes after treatment were measured using flow cytometry to elucidate the antitumor mechanism of SCH772984. In the spleen of the corn oil group, the percentages of CD4^+^CD69^+^ lymphocytes and CD8^+^CD69^+^ lymphocytes were 14.50% (**Figure 4A**) and 10.76% (**Figure 4B**), respectively. In the spleen of the SCH772984 group, the percentages were 25.00% and 18.95%, respectively. In addition, the percentages of CD4^+^CD69^+^ lymphocytes and CD8^+^CD69^+^ lymphocytes in lymph nodes from the corn oil group were 13.70% (**Figure 4C**) and 8.52% (**Figure 4D**), respectively. The percentages in the SCH772984 group were 17.87% and 13.73%, respectively. The numbers of CD4^+^CD69^+^ and CD8^+^CD69^+^ cells increased in the spleen and lymph nodes of mice in the SCH772984 group.

## Discussion

Of the two categories of MDSCs, PMN-MDSCs are more prevalent in tumor-bearing mice, while M-MDSCs show greater immunosuppressive properties (27–29). M-MDSCs express monocyte biomarkers such as CD115 and F4/80, which suppress CD8^+^ T cells and result in immunosuppression (27, 30, 31). Thus, MDSCs have been recognized as an important therapeutic target in tumor treatment. In a clinical study, the combined use of cytokine-induced killer cells, gemcitabine and 5-fluorouracil decreased the frequency of MDSCs and increased the survival of patients with metastatic renal cell carcinoma and pancreatic cancer (32). In another study, MDSCs were targeted with inhibitors of tumor necrosis factor-related apoptosis-induced ligand receptors (TRAIL-R) and some benefits were observed (33). However, the systemic administration of these drugs can not eliminate MDSCs effectively. Recently, an inhibitor of p-STAT3 only eliminated MDSCs in the peripheral tissues, such as the spleen, while MDSCs in tumors were not affected (19). Indeed, in the present study, our results revealed the gene expression of MDSC derived from tumor was significantly different from that of MDSC derived from spleen from the same tumor-bearing mouse. The treatment of MAPK inhibitors resulted in the decrease of MDSCs both *in vivo* and *in vitro*. Subsequently, our data showed that intratumor injection of ERK inhibitor SCH772984 led to macrophage polarization, enhancing T cell response and exerting an anti-tumor effect.

The MAPK pathway participates in oncogenesis, tumor progression and metastasis (34–37). Previous studies suggested that activation of MAPK participated in promoting bladder tumorigenesis (38). Additionally, the activated MAPK pathway participates in the progression of colorectal cancer (39). Since increased numbers of MDSCs play an important role in tumor growth and a significant change in MAPK activity in MDSCs from the tumor site has been observed, a reasonable hypothesis is that MDSCs promote tumor growth by activating MAPK and that MDSCs might be targeted by MAPK inhibitors. In this study, we examined RNA sequencing data from MDSCs derived from the spleen and tumor using the magnetic bead separation technique. Our results showed that MAPK activity was increased in tumor-derived PMN-MDSCs and M-MDSCs. Three major MAPK pathways, including ERK, p38 and JNK, were studied to analyze the role of MAPKs in MDSCs. SCH772984 and SP600125 significantly increased MDSC apoptosis *in vitro*. In addition, intratumor injection of SCH772984 dramatically result in tumor regression.

As mentioned above, ERK is an important member of the MAPK family that participates in apoptosis. Inhibition of ERK or prevention of ERK phosphorylation increases cell apoptosis (40, 41). ERK inhibitors also affect the tumor microenvironment by increasing the infiltration of lymphocytes and macrophages (41). In this study, SCH772984 treatment not only induced the apoptosis of MDSCs, but also significantly decreased the number of F4/80^+^CD206^+^ TAM which are a key regulatory factor in the tumor microenvironment affecting the therapeutic response, while M1-like macrophages increased. In the meantime, the increases in the percentages of CD4^+^ and CD8^+^ T cell, CD4^+^CD69^+^ and CD8^+^CD69^+^ T cell were found in tumor tissue and in lymph node near the tumor. These results suggested that ERK 1/2 inhibition could reverse the immunosuppressive state of tumor microenvironment and improve the T cell response.

Generally, intravenous injection of SCH772984 is a commonly used strategy for ERK inhibition. However systemically administration was limited by systemic toxicity and other side effects. On the contrary, intratumor injection might minimize drug dose, reduce systemic side effects and optimize distribution of drug to the target site (42). Importantly, intratumoral therapy could alter the tumor microenvironment from immunosuppressive to immunostimulatory and generate systemic T cell responses. There are some clinical trials using intratumoral delivery system. In our study, the intratumor injection of the drug at a smaller dose than systemic treatment inhibited tumor growth and affected the tumor microenvironment. The methods and results of our study provided experimental basis for the treatment of tumor with MAPK inhibitors such as SCH772984. However, further studies should be performed to confirm its translational value and the potential clinical applicability. In conclusion, we confirmed that inhibited the ERK/MAPK pathway could suppress tumor growth and affected the tumor microenvironment by promoting MDSC apoptosis. Strategies targeting MAPK in MDSCs might be a novel therapeutic approach to tumor treatment.

## Data Availability Statement

The datasets presented in this study can be found in online repositories. The names of the repository/repositories and accession number(s) can be found in the article/supplementary material.

## Ethics Statement

The animal study was reviewed and approved by the ethics committee of West China Hospital of Stomatology, Sichuan University.

## Author Contributions

Conception and design: BS and XW. Acquisition of data: JY and HL. Analysis and interpretation of data: JY, WL, and HL. Drafting the manuscript: JY and HL. Critically revising the manuscript: BS. Providing final approval of the version to be published: BS and XW. All authors contributed to the article and approved the submitted version.

## Funding

This study was funded by the National Natural Science Foundation of China, Grant/Award Numbers: 81702271 and 81972193; the Scientific Research Foundation for Recruited Talents, West China Hospital of Stomatology Sichuan University, Grant/Award Number: QDIF2019-1; the China Postdoctoral Science Foundation, Grant/Award Number: 2019M650246, 2020T130448; and the Department of Science and Technology of Sichuan Province, Grant/Award Number: 2019YJ0041; Postdoctoral Research and Development Fund of West China Hospital of Sichuan University, Grant/Award Number: 2020HXBH060.

## Conflict of Interest

The authors declare that the research was conducted in the absence of any commercial or financial relationships that could be construed as a potential conflict of interest.
